# Persimmon Fiber-Rich Ingredients Promote Anti-Inflammatory Responses and the Growth of Beneficial Anti-Inflammatory Firmicutes Species from the Human Colon

**DOI:** 10.3390/nu16152518

**Published:** 2024-08-01

**Authors:** Lucía López-Bermudo, Bryan Moreno-Chamba, Julio Salazar-Bermeo, Nicholas J. Hayward, Amanda Morris, Gary J. Duncan, Wendy R. Russell, Antonio Cárdenas, Ángeles Ortega, Blanca Escudero-López, Genoveva Berná, Nuria Martí Bruña, Sylvia H. Duncan, Madalina Neacsu, Franz Martin

**Affiliations:** 1 Andalusian Center of Molecular Biology and Regenerative Medicine (CABIMER), Pablo de Olavide University, University of Seville, CSIC, 41092 Seville, Spain; lucia.lopez@cabimer.es (L.L.-B.); maortega@upo.es (Á.O.); besclop@upo.es (B.E.-L.); gberamo@upo.es (G.B.); 2 Biomedical Research Network on Diabetes and Related Metabolic Diseases (CIBERDEM), Instituto de Salud Carlos III, 28029 Madrid, Spain; 3 Institute of Research, Development and Innovation in Sanitary Biotechnology of Elche, Miguel Hernández University, 03202 Elche, Spain; bryan.morenoc@umh.es (B.M.-C.);; 4 Institute of Food Engineering for Development, Universitat Politècnica de València, 46022 Valencia, Spain; 5 Rowett Institute, University of Aberdeen, Foresterhill, Aberdeen AB25 2ZD, UK

**Keywords:** dietary fiber, *Faecalibacterium prausntizii*, *Eubacterium eligens*, pectins, anti-inflammatory activity, butyrate

## Abstract

Persimmon fruit processing-derived waste and by-products, such as peels and pomace, are important sources of dietary fiber and phytochemicals. Revalorizing these by-products could help promote circular nutrition and agricultural sustainability while tackling dietary deficiencies and chronic diseases. In this study, fiber-rich fractions were prepared from the by-products of Sharoni and Brilliant Red persimmon varieties. These fractions were quantified for their phenolic composition and assessed for their ability to promote the growth of beneficial human colonic Firmicutes species and for their in vitro anti-inflammatory potential. Gallic and protocatechuic acids, delphinidin, and cyanidin were the main phenolics identified. *Faecalibacterium prausnitzii* strains showed significantly higher growth rates in the presence of the Brilliant Red fraction, generating more than double butyrate as a proportion of the total short-chain fatty acids (39.5% vs. 17.8%) when compared to glucose. The fiber-rich fractions significantly decreased the inflammatory effect of interleukin-1β in Caco-2 cells, and the fermented fractions (both from Sharoni and Brilliant Red) significantly decreased the inflammatory effect of interleukin-6 and tumor necrosis factor-α in the RAW 264.7 cells. Therefore, fiber-rich fractions from persimmon by-products could be part of nutritional therapies as they reduce systemic inflammation, promote the growth of beneficial human gut bacteria, and increase the production of beneficial microbial metabolites such as butyrate.

## 1. Introduction

Persimmon (*Diospyros kaki* Thunb.), belonging to the Ebanaceae family, is a fruit that originated in China [1]. Due to its taste, nutrient content, bioactive compounds, and health benefits [2], its consumption has increased in recent years, with more than 4 million tons/year produced [3] in countries like China, South Korea, Japan, and Spain. The astringent varieties like ‘Red Brilliant’ and ‘Triumph or Sharoni’ are the most commercially cultivated [4], in Spain and Israel [5]. The persimmon processing leads to the generation of several by-products, such as peels, pomace, hulls, and leaves [6], which could deliver zero-waste persimmon production, promoting circular nutrition [7]. Furthermore, the development of nutraceuticals and functional food ingredients could tackle global dietary deficiencies/malnutrition and diseases by delivering important nutrients like dietary fiber and bioactive phytochemicals [8,9]. 

The main waste produced by persimmon industrialization is the peel and pulp, which are separated during juice production [10], which serves as a potential valuable source for phytochemicals and polysaccharides. Persimmon by-products are rich in carotenoids such as lutein, zeaxanthin, β-cryptoxanthin, β-carotene, α-carotene, and lycopene, as well as polyphenols like gallic acid, fumaric acid, epigallocatechin, and catechin [11,12]. Although some research has been carried out studying the bioactive composition of persimmon by-products [1], bonded phenolics and carotenoids to dietary fiber and indigestible compounds hold promise to be fully explored and utilized. 

Persimmon by-product processing involves different methods, such as the use of solvents or the application of technologies such as ultrasound, to obtain bioactive compounds. Fermentation, an ancient technology, has been applied for shelf-life enhancement, organoleptic improvement, and the production of certain metabolites [13,14]. It also shows potential for bioactive extraction from food materials, influencing the discovery of new bioactive compounds, the improvement of health benefits, and even an increase in the bioaccessibility and bioavailability of some compounds [13,14], which is a key aspect during the gastrointestinal digestion of complex macromolecules like dietary fibers. 

The dietary fibers consumed in our diets usually escape digestion by bacteria in the upper intestinal tract and are then colonized by bacteria in the colon [15]. The human colon hosts a dense and varied collection of microorganisms. Molecular profiling of the gut microbiota shows that the healthy human colon hosts two bacterial phyla, the Firmicutes and Bacteroidetes, usually making up the majority of the total microbiota [16] and the composition and metabolic output of the human gut microbiota have a major impact on health and disease [17]. Both the micronutrient and macronutrient content of diets, including fiber content and composition, are important drivers in modulating the composition of the gut microbiota and activities [18], some of which can have an impact on wider health, including the brain [19]. 

Certain colonic anaerobes possess key fiber degrading enzymes [15] and cross feed to other members of the microbiota in the large intestine [20]. Moreover, primary and secondary metabolites produced by the fermentation of selective compounds in dietary fiber may lead to the stimulation of anti-inflammatory response pathways, playing a role in the modulation of severe gastrointestinal (GI) disorders like ulcerative colitis [21]. Furthermore, the interaction of dietary polysaccharides and gut microbiota supports the modulation of the gut barrier, which is associated with a stimulation of the immune system and a reduction in pro-inflammatory cytokines, especially in the exposure to external molecules like pathogenic bacteria or their endotoxins [22]. In this sense, dietary fiber, throughout its interaction with gut microbiota, can be implied in the modulation of the cellular adaptative, increasing its importance to be consumed. Through their receptors in the immune cells, metabolites originating from the gut microbiota influence the metabolism of the immune cells. These compounds from various microbes have an anti-inflammatory activity. Gut microbiota-derived metabolites can inhibit inflammatory cells and improve the differentiation and activity of immunosuppressive cells [23]. Persimmon tannins [24] and polysaccharides [25] modify the microbiota balance in rats and mice and act as anti-inflammatory and antioxidant agents [26]. 

The average intake of fiber in the UK is 10–15 g despite the recommendation to maintain health being 30 g fiber per day [27]. Plant cell walls are a valuable source of carbon for gut bacteria and are primarily composed of cellulose, hemicellulose, and pectin. Pectins are structurally highly complex, and degradation of pectin-rich fibers requires that pectin degraders possess an array of glycosyl hydrolase and polysaccharide lyases [28]. One Firmicutes species, *Eubacterium* (now *Lachnospira*) *eligens*, has been reported to have a wide-ranging collection of specialist pectin-degrading enzymes and has been reported to be efficient at degrading pectins whilst other species, such as *Faecalibacterium prausnitzii* which is one of the most abundant bacteria in a healthy human colon, is likely to be more specialized and possess the ability to utilize more specific components of pectin [28,29].

The main products formed by fermentation by the human gut microbiota are the short-chain fatty acids, including the three main acids acetate, propionate, and butyrate. In particular, the latter product, butyrate, is the main end product of *Faecalibacterium prausnitzii* and is generated by these bacteria utilizing acetate, generated by certain bacteria, such as *Eubacterium eligens*, to form butyrate via the butyryl CoA:acetate CoA transferase route. Butyrate is the major energy source for colonocytes, possesses anti-inflammatory activity, and regulates apoptosis [30]. Metabolism of dietary fibers also results in the release of plant polyphenols, which are largely responsible for giving fruits and vegetables their color and can also have potent anti-inflammatory activities [31]. 

This study aims to revalorize persimmon by-products by producing fiber-rich ingredients, characterize their phenolic composition, and assess their ability to promote beneficial, understudied bacteria, in particular representative Firmicutes species, from the human colon. Furthermore, the study assessed the in vitro anti-inflammatory potential of the persimmon fiber-rich ingredients.

## 2. Materials and Methods

### 2.1. Preparations of Soluble Fiber-Rich Fractions from the Persimmon Fruits

**Figure 1:**
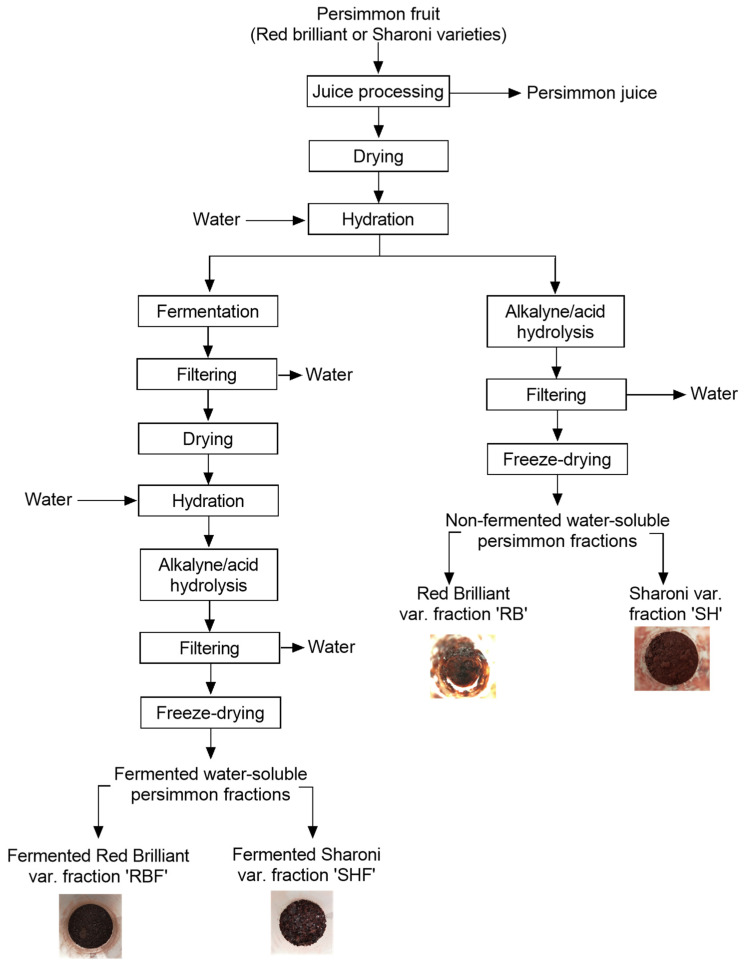
Diagram for processing of the persimmon fruit by-products to obtain soluble fiber-rich fractions through alkaline/acid hydrolysis or fermentation.

Two varieties of fresh persimmon fruits (Red Brilliant and Sharoni varieties) were purchased from a local market in Elche, Spain. The fruits were washed and cut into 2–3 cm wedges to be further processed using an industrial fruit juice machine to separate juice and solid by-products. The by-products, made up of pulp and peels, were vacuum dried at 60 °C and stored at 20 °C for further treatment (Figure 1). Batches of the by-product of each persimmon variety were hydrated with water (1:10 *w*/*v*) at room temperature prior to fermentation or hydrolysis treatments. 

Fermentation treatment: A total of 1 L of each suspension containing by-products from each variety was inoculated with 1 mL of fresh suspensions (10^7^ CFU/mL) of each *Streptococcus salivarius* subsp. *thermophilus* CECT 7207 and *Lactobacillus casei* CECT 475. The suspensions were incubated at 37 °C for 24 h at 150 rpm in a benchtop incubator shaker. After incubation, the suspensions were filtered, discarding the liquid. The solid fractions were vacuum dried at 60 °C overnight. The powder obtained from each sample was further subjected to hydrolysis. 

Hydrolysis treatment: Each suspension containing by-products from each variety, fermented and non-fermented, was exposed to alkaline hydrolysis by adjusting the pH of each suspension to 12 with 5 M NaOH (color change of suspension from clear to deep dark) using a pH meter (Hanna Instruments edge^®^, Daselab S.L., Valencia, Spain). The mixtures were heated at 40 °C for 24 h under constant shaking (150 rpm) in a benchtop incubator shaker; then, the pH was lowered to 2.5 with 5 M HCl (acidic hydrolysis, color change from deep dark to red). The suspensions were filtered, and the liquid fractions were freeze dried (LyoQuest, Telstar, Barcelona, Spain) to obtain persimmon water-soluble fractions of Red Brilliant (RB) and Sharoni (SH) without fermentation, and water-soluble fermented persimmon fractions of Red Brilliant (RBF) and Sharoni (SHF).

### 2.2. Chemicals and Reagents 

Standards and reagents: Standards for the free sugar analysis, including glucose, fructose, sucrose, rhamnose, fucose, arabinose, xylose, mannose, galactose, galacturonic acid, glucuronic acid, and maltose, were purchased from Sigma-Aldrich (Dorset, UK) and Thermo-Fisher Scientific (UK). Standards for the anthocyanin aglycones (anthocyanins) analysis, including delphinidin (>95%), cyanidin (>95%), pelargonidin (undeclared purity), and peonidin (>96.5%), were all purchased from Sigma-Aldrich (Dorset, UK) and malvidin (>95%) from Phytolab, Germany. The aglycone standard, petunidin, was purchased from ChemFaces (Wuhan, China) at a purity of >95%. All the phenolic standards were purchased from Sigma-Aldrich (Gillingham, UK), Phytolab (Vestenbergsgreuth, Germany) or synthesized, as described previously [32]. General reagents were purchased from Sigma-Aldrich (Dorset, UK) and Fischer Scientific (Loughborough, UK). Reagents used for hydrolysis to obtain persimmon fiber-rich fractions were purchased from Sigma-Aldrich (Madrid, Spain), while strains used to ferment persimmon by-products to obtain fermented persimmon fractions were purchased from Spanish Type Culture Collection (CECT, Valencia, Spain).

### 2.3. Analysis of Free Sugars Composition of Persimmon Fiber-Rich Fractions

The quantification of mono- and disaccharides from the persimmon fraction was performed using a 1260 Infinity HPLC from Agilent (Wokingham, UK) equipped with a RI detection and an Asahipak NH2P-50 4E (5 μm; 25 cm × 0.46 cm), (Shodex, Japan) column connected to an Asahipak NH2P-50G 4A pre-column (4.6 mm × 10 mm, Shodex, Japan). Persimmon fractions (approx. 0.05 g, n = 3) were dissolved in 250 μL of water and filtered using 0.2 µm filters prior to the HPLC analysis using an isocratic solvent program consisting of 70% acetonitrile at a constant flow of 1 mL/min. Quantification of the free sugars was performed using external calibration curves using validated standards for each sugar analyzed.

### 2.4. Analysis of Anthocyanin Composition of Persimmon Fiber-Rich Fractions

Quantification of the anthocyanidin (anthocyanin aglycones) content from persimmon fiber-rich fractions was performed by extraction and hydrolysis methods adapted from Zhang et al. [33]. Briefly, samples of persimmon (0.05 g, n = 3) were extracted with methanol–water–hydrochloric acid (ratio of 50:33:17; *v*/*v*/*v*; 3 mL) three consecutive times, and the supernatants and the pellet were combined and hydrolyzed at 100 °C for 60 min. Hydrolyzed samples were then immediately cooled to room temperature, filtered using 0.2 µm filters, and analyzed using a 1260 Infinity HPLC from Agilent (Wokingham, UK) with a Synergi 4 µm Polar-RP 80A (250 × 4.6 mm) column with a Polar-RP 4 × 3 mm pre-column from Phenomenex (Macclesfield, UK). The HPLC system was equipped with a DAD detector, and spectra were recorded between 200 and 700 nm. The chromatograms were monitored at 530 nm. For the HPLC separations, the following solvents were used: A: formic acid (2.125%) and B: acetonitrile/methanol (85:15, *v*/*v*) in an isocratic program using 18% B for 40 min at 1 mL/min, as described by Zhang et al. [33]. The column temperature was held at 35 °C. The separation and quantification of anthocyanins were performed using external standardization, as described previously [34]. 

### 2.5. Analysis of Other Phenolic Molecules Composition of Persimmon Fiber-Rich Fractions

Phenolic molecule extraction used an existing method [35], which has been further amended. Briefly, the persimmon samples (approx. 0.1 g dry weight; n = 3) were suspended in HCl (0.2 M; 3 mL) and then extracted three consecutive times into ethyl acetate (5 mL). The three ethyl acetate extracts were combined and dried before being dissolved with methanol (0.5 mL), which represented the “free fraction” and was stored at −70 °C prior to LC-MS analysis.

The remaining aqueous fraction’s pH was adjusted for the alkaline hydrolysis using NaOH (4 M) and stirred at room temperature for 4 h under nitrogen. The pH was reduced afterwards to 2 with HCl (6 M), and samples were extracted again into ethyl acetate (3 × 5 mL). The three ethyl acetate extracts were combined and dried before being dissolved with methanol (0.5 mL), which represented the ‘alkaline-bound fraction’.

The pH of the aqueous fraction was then adjusted again using HCl (6M) for the acid hydrolysis and incubated at 95 °C for 30 min. The samples were cooled to room temperature and extracted three times with ethyl acetate (5 mL each). The three ethyl acetate extracts were combined and dried being dissolved with methanol (0.5 mL), which represented the ‘acid-bound fraction’. The alkaline-bound and acid-bound fractions were combined and represented as bound fractions analyzed using LC-MS/MS analysis. 

To prepare samples for LC-MS analysis, an aliquot of free and respectively bound fractions dissolved in methanol, as described above, was mixed with Internal Standard 1 (IS1) for negative-mode mass spectrometry (13C benzoic acid) and Internal Standard 2 (IS2) for positive-mode mass spectrometry (2-amino-3,4,7,8-tetramethylimidazo [4,5-ƒ]quinoxaline).

The liquid chromatography separation of phenolic metabolites was performed on an Agilent 1100 LC-MS system using a Zorbax Eclipse 5 µm, 150 mm × 4 mm column from Agilent Technologies (Wokingham, UK) as described elsewhere [36]. All persimmon sample extracts prepared as mentioned above were screened for phenolic acids and derivatives, flavonoids, and lignan metabolites. Three gradient solvents were used to separate the different categories of metabolites, with the mobile-phase solvents being water containing 0.1% acetic acid (A) and acetonitrile containing 0.1% acetic acid (B). In all cases, the flow rate was 300 μL/min with an injection volume of 5 μL. The LC eluent was directed, without splitting, into an ABI 3200 triple-quadrupole mass spectrometer (Applied Biosystems, Warrington, UK) fitted with a turbo-ion spray source. All the metabolites were quantified using multiple reaction monitoring. 

### 2.6. Preparation of Bacterial Cultures and Assessment of Growth on Persimmon Fiber-Rich Fractions Using a Microtitre Plate Assay 

Six bacterial strains were tested for their ability to utilize soluble persimmon fiber-rich fractions as growth substrates using the microtiter plate method in triplicate (technical replicates) as described previously [28]. 

Bacterial strains included two strains that represented the two phylogroups of *Faecalibacterium prausnitzii* (A2–165, M21/2), *Coprococcus species* L2-50 held by the Rowett Institute, Aberdeen; *Eubacterium eligens* (DSM3376 = ATCC27750); and *Bifidobacterium bifidum* (DSM20456) were purchased from Deutsche Sammlung von Mikroorganismen und Zellkulturen (DSMZ) (Braunschweig, Germany); and *Bacteroides thetaiotaomicron* (ATCC 5482) was from the American type culture collection (ATCC) (Manassas, VA, USA). The strains for growth studies were pre-prepared by culturing on M2 medium containing 0.2% (*w*/*v*) of each glucose, cellobiose, and soluble potato starch [37] for 20–24 h at 37 °C under a stream of CO_2_ gas. Sterile microtiter plates were maintained in an anaerobic cabinet (Don Whitley, Shipley, UK) for 24 h prior to adding 200 μL of anaerobic pre-reduced basal M2 medium containing either 0.2% (*w*/*v*) of glucose or persimmon fractions as the single growth substrate. The wells were then inoculated with overnight cultures grown anaerobically in M2GSC medium in triplicate for each substrate or basal medium as a control. The plates were covered with Q-optical seals (Bio-Rad, UK), and then the plates were incubated for 24 h at 37 °C in a Tecan Safire 2 microplate reader (Tecan Group Ltd., Männerdorf, Switzerland), with optical readings at 650 nm taken every hour following low-speed shaking for 5 s, as described previously [28]. For the mixed bacterial culture studies and assessment of growth on persimmon fibers using a microtiter plate assay, the strains were prepared and mixed in equal amounts and processed, as described. 

### 2.7. Growth Rate Determinations

Growth rate calculations were performed using at least three values during the mid-exponential growth phase, as described previously [38]. Briefly, semi-log values of the growth from the microtiter plates were used to determine the growth rates per hour.

### 2.8. Quantification of Short-Chain Fatty Acid Analysis 

Shor- chain fatty acid (SCFA) formation was determined in culture supernatants by gas chromatography, as described previously [39]. Briefly, following derivatization of the samples using N- tert-butyldimethylsilyl-N-methyltrifluoroacetamide, the samples were analyzed using a Hewlett–Packard gas chromatograph fitted with a fused silica capillary column with helium as the carrier gas. The lower detection limit for each of the acids was 0.2 mM.

### 2.9. Mammalian Cell Culture

The cell lines human colorectal adenocarcinoma (Caco-2), human hepatoma (HepG2), murine macrophage (RAW 264.7), and 3T3-L1 mouse embryo fibroblast were obtained from ATCC (American Type Culture Collection, Gaithersburg, MD, USA).

All cell lines were usually grown in culture T-flasks in a CO_2_-incubator at 37 °C with 5% CO_2_. The culture medium was Dulbecco’s Modified Eagle Medium (DMEM) (Sigma Aldrich, Madrid, Spain) supplemented with 0.1 mM of non-essential amino acids (Gibco, Thermo Fisher Scientific Inc., Waltham, MA, USA), 100 U/mL of penicillin/0.1 g/mL of streptomycin (Gibco), 10% fetal bovine serum (FBS) (Hyclone Laboratories Inc., Logan, UT, USA), and 2 mM L-glutamine (Gibco). The culture medium was refreshed every 24–48 h. At a confluency of 70–80% (every 2–3 days), cells were split at a ratio of 1:3–1:5 using trypsin-EDTA (Gibco). For all the experiments, cell passages 10–30 were used.

In the case of 3T3-L1, they were maintained in a pre-adipocyte state using the above-mentioned splitting and feeding protocol. To obtain the adipocyte phenotype, cells were cultured to 85–90% confluency and maintained for 3 days in the previously indicated feeding media. Then, the medium was completely changed to the differentiation media. This medium included DMEM supplemented with 10% FBS, 1 µg/mL of insulin (Sigma Aldrich, Madrid, Spain), 0.25 µM dexamethasone (DEX) (Sigma-Aldrich, Madrid, Spain), and 0.5 mM 3-Isobutyl-1-methylxanthine (IBMX) (Sigma-Aldrich). This medium was left on the cells for 48 h. Afterwards, the cells were cultured for another 5 days in post-differentiation media containing insulin without DEX and IBMX. Fresh insulin was added to media each day of feeding. This post-differentiation medium was added to the cells until the end of the experiment. Adipocytes were used 7–10 days after differentiation.

### 2.10. Persimmon Fiber-Rich Fractions Preparation for Cell Culture Studies

Individual stock solutions of the four fractions (RB, RBF, SH, and SHF) at 250 mg/mL were prepared in dimethyl sulfoxide (DMSO) used as a vehicle (Thermo Fisher Scientific Inc.). Different concentrations of the stock solutions were prepared in the corresponding media (100, 250, and 500 μg/mL), with the final DMSO concentrations being 0.04%, 0.1%, and 0.2%, respectively. A concentration of 10% (*v*/*v*) DMSO was used as a control for the maximal cytotoxicity.

### 2.11. Viability and Cytotoxicity Assays

Cells were seeded on a 96-well plate (Thermo Fisher Scientific Inc.) at 5 × 10^4^ (HepG2), 2 × 10^4^ (Caco-2), 10 × 10^3^ cells/well (adipocytes derived from 3T3-L1), and 10 × 10^3^ cells/well (RAW 264.7) and incubated for 24 h at 37 °C and 5% CO_2_. The cell culture medium was then removed, and the cells were exposed, for 24 h and 48 h, to 100 μL of cell culture medium (control), 10% DMSO in medium, the three different concentrations of the four fractions (100, 250, and 500 μg/mL) in the complete medium, or different DMSO concentrations equivalent to the amount of vehicle in the corresponding assayed fractions (0.04%, 0.1%, and 0.2%). Empty wells and sample controls, containing the medium with no cells to determine the baseline signal, were included in all assays.

For the human cell lines HepG2 and Caco-2, the Alamar Blue assay was used to determine the effect of the extracts on cell viability. This assay incorporates an oxidation–reduction (REDOX) indicator, assessing the mitochondrial ability to reduce resazurin into the fluorescent product resorufin [40]. Briefly, 100 μL of Alamar Blue working solution was prepared by mixing cell culture medium with a stock solution of resazurin sodium salt (5 mg/mL) (Panreac AppliChem, Barcelona, Spain) in phosphate-buffered saline (PBS) in a 10:1 ratio and then added to each well. Cells were then incubated for 2 h at 37 °C before measuring fluorescence.

For adipocytes and RAW 264.7 cells, the Live/Dead Viability/Cytotoxicity Kit for mammalian cells (Thermo Fisher Scientific Inc.) was used. This 2-color fluorescence cell viability assay is based on 2 probes: calcein AM, which identifies live cells, and ethidium homodimer-1 (EthD-1), which detects dead cells. Consequently, live cells are stained green and dead cells are stained red. The cell viability and cytotoxicity were evaluated in line with the manufacturer’s instruction. Briefly, after incubations with the different concentrations of the four fractions, the supernatant was removed, and the cells were washed with PBS twice. Moreover, samples of live and dead cells were prepared for controls. To prepare dead cell controls, live cells were treated with 0.1% saponin for 10 min. The following controls were prepared: (i) all live cells labeled only with EthD-1; (ii) all live cells labeled only with calcein AM; (iii) all dead cells labeled only with EthD-1; (iv) all dead cells labeled only with calcein AM; (v) one cell-free control labeled with EthD-1; and another cell-free control labeled with calcein AM. After determining the optimal dye concentrations, cell culture medium with calcein AM (2 uM) and EthD-1 (4 uM) was added to each well and incubated for another 30 min. The supernatant was then removed, and cells were washed with PBS twice. Live and dead cell numbers were calculated from standard curves with known numbers of live and dead cells. 

The results were averaged over 4 different independent experiments with 5 replicates per experiment. Fluorescence was measured with a multimodal Varioskan Lux spectrophotometer (Thermo Fisher Scientific Inc.). For the alamar blue assay, the excitation and emission wavelengths were 530 and 590 nm, respectively. For the Live/Dead kit, the excitation and emission filters were 485/530 nm (live cells) and 530/645 nm (dead cells). The results indicated the percentage of viable cells in relation to the control of each compound after background fluorescence was subtracted. This was calculated as the number of live cells divided by the number of total cells at each time point.

### 2.12. Inflammatory Cytokine Tests 

Caco-2 and RAW 264.7 cells were seeded in 12- and 24-well plates at a density of 2 × 10^5^ and 10 × 10^3^ cells/well, respectively, and allowed to adhere for 22 h before initiating different treatments. To analyze the anti-inflammatory effect of the fractions, inflammation was induced by treating Caco-2 cells with 25 ng/mL interleukin 1β (IL-1β) (R&D Systems, Inc., Minneapolis, MN, USA) and RAW 264.7 cells with 1 µg/mL lipopolysaccharide (LPS) (Sigma-Aldrich). IL-1β and LPS were diluted in complete media from a 10 μg/mL stock and 1 mg/mL stock solution in PBS, respectively. 

The protocol was to culture the cells with media containing 100 μg/mL of the different four fractions (100 μg/mL) for 2 h at 37 °C. Next, cells were cultured with the inflammatory stimulus for 24 h in the presence or absence of the same concentrations of the different four fractions in the completed media. Non-stimulated Caco-2 and RAW 264.7 cells were cultivated in parallel with the four different fractions (100 μg/mL) in complete media for 24 h and used as controls. Additional controls were cells cultivated for 24 h with complete media, media with vehicle (DMSO 0.04%), or media with IL-1β (25 ng/mL) or LPS (1 µg/mL). After treatments, the extracellular media were collected, centrifuged at 1500 rpm to remove debris, and kept at −20 °C until analysis. 

Interleukin 6 (IL-6) and tumor necrosis factor α (TNF-α) were quantified using a sandwich ELISA method (R&D Systems, Inc.) according to the manufacturers’ instructions. Fluorescence values were recorded with a microplate reading Varioskan Lux spectrophotometer (Thermo Fisher Scientific Inc.) at 450 nm for IL-6 and 520 nm for TNF-α.

### 2.13. Cellular Antioxidant Enzyme Activity Assays 

HepG2 cells were seeded in 12-well plates at a density of 1 × 10^6^ cells/well in complete medium and allowed to adhere for 24 h before initiating different treatments. After incubating cells for 24 h at 37 °C with each of the four different fractions (100 μg/mL) in complete media, oxidative stress was induced by cultivating the cells with 500 μM H_2_O_2_ for another 2 h at 37 °C, either in the presence or absence of the same concentrations of the different four fractions in complete media. Cells cultivated in parallel with the four different fractions (100 μg/mL) in complete media were used as controls. Cells incubated in complete media and then added 500 μM H_2_O_2_ for 2 h at 37 °C were treated as the model group. Additional controls were cells cultivated in complete media or in media with vehicle (DMSO 0.04%).

After treatment, cells were then lysed in an ice-cold solubilization buffer (10 mM Tris ClH pH 7.5, 150 mM ClNa, 0.1 mM EDTA) and a supernatant was collected by centrifuging at 10,000× *g* for 15 min at 4 °C. Samples were frozen at −20 °C until the enzyme activity assay.

Superoxide dismutase (SOD), catalase (CAT), and glutathione reductase (GR) activities were determined using colorimetric and fluorescent activity kits (Thermo Fisher Scientific Inc.) according to the manufacturers’ instructions. Lectures were recorded with a microplate reading Varioskan Lux spectrophotometer (Thermo Fisher Scientific Inc.) at 450 nm for SOD, 560 nm for CAT, and 390 nm excitation and 510 nm emission for GR. Results were expressed as U/mL for SOD and CAT and mU/mL for GR.

### 2.14. Statistical Analysis

All the analyses were performed in triplicate, and the results are presented as the mean ± standard deviation (SD). The significance differences between persimmon sample composition (in terms of varieties and preparation treatments) were assessed using a paired *t* test (significance level *p* < 0.05) with a significance threshold of * *p* < 0.05, ** *p* < 0.01, and *** *p* < 0.001. The phytochemicals profiles measured by LC-MS/MS were determined by principal component analysis (PCA), unit variance (UV)-scaled using SIMCA 14.1 (Umetrics, Cambridge, UK). For the rest of the results presented, statistical comparisons were performed using the two-tailed unpaired *t* test, U-Mann–Whitney test, and the Kruskal–Wallis test. *p*-values less than 0.05 were considered statistically significant. 

The SPSS Version 29.0 software package (IBM, Armonk, NY, USA) was used for statistical analysis. GraphPad Prism 10.0 (GraphPad Software Inc, San Diego, CA, USA) was used to graph the data. 

## 3. Results

The persimmon fruit by-products from varieties ‘Red Brilliant’ and ‘Sharoni’ were further processed to obtain soluble fiber-rich fractions (Figure 1) to determine their potential functionalities. 

### 3.1. Composition of the Soluble Persimmon Fiber-Rich Fractions

The free monosaccharides compositions of Brilliant Red (RB), Sharoni (SH), RBF, and SHF fractions are shown in Table 1. RB fraction had significantly (*p* < 0.05) higher levels of fructose and glucose (*p* < 0.001) than SH fraction. Moreover, as might be expected, fermented fractions had significantly (*p* < 0.001) lower levels of fructose and glucose than non-fermented fractions.

**Figure 2:**
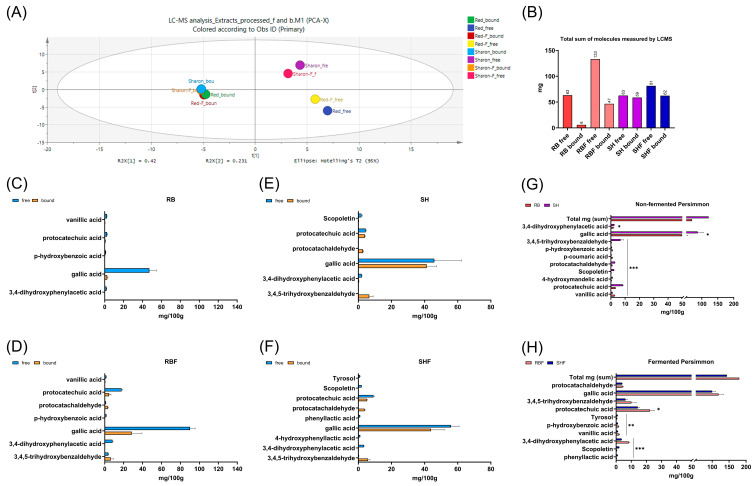
Composition of the soluble persimmon fiber-rich fractions. (**A**) The principal component analysis (PCA) of all plant metabolites measured by LCMS in free and bound fractions from persimmon samples. (**B**) The total phytochemicals (free and bound) content (in mg) obtained by summing the individual plant metabolites measured by LCMS. (**C**–**F**) The most abundant molecules (above 1 mg/100 g), measured by LCMS in free and bound fractions (as mean ± SD, n = 3) in the persimmon fiber-rich fractions. (**G**,**H**) Ten most abundant plant metabolites (as mean ± SD, n = 3 summing the bound and free extractable molecules) measured in the persimmon samples, where RB and RBF are fiber-rich persimmon fractions from Brilliant Red variety, fermented (F) or not; respectively, SH and SHF are fiber-rich persimmon fractions from Sharoni variety, fermented (F) or not; * *p* < 0.05, ** *p* < 0.01, *** *p* < 0.001. RB: Red Brilliant; RBF: Red Brilliant fermented; SH: Sharoni; SHF: Sharoni fermented.

Principal component analysis (PCA) analysis of the persimmon metabolites measured by LCMS showed that the phytochemicals profile of the molecules extracted in the free fractions of the RB and RBF was different than the one from SH and SHF. Furthermore, this analysis also indicates that the profile of the bound phytochemicals, those extracted following the hydrolysis (alkaline and acid), is also different that the one extracted in the free fraction, but the profile of the bound phytochemicals for RB, RBF, SH, and SHF is similar (Figure 2A). 

Gallic acid and protocatechuic acid were the most abundant molecules measured by LC-MS/MS in the persimmon samples, they are mostly found in free forms in the RB and RBF and more equally distributed between free and bound forms in the SH and SHF samples (Figure 2C–F). The fermentation had a significant effect on the extractability of the phytochemicals, especially for the Brilliant Red variety of persimmon, incrementing the number of molecules extractable (Figure 2B–D); the sum of free molecules measured by LCMS in RB before fermentation rises from 63 mg to 133 mg after the fermentation, and the bound molecules from 6 to 47 mg, respectively. The Sharoni variety seems to be richer in the phytochemicals measured by LCMS than the Brilliant Red variety; however, as the fermentation increases the extractability of the molecules, the RBF fraction is richer in extractable phytochemicals (Figure 2G,H).

**Figure 3:**
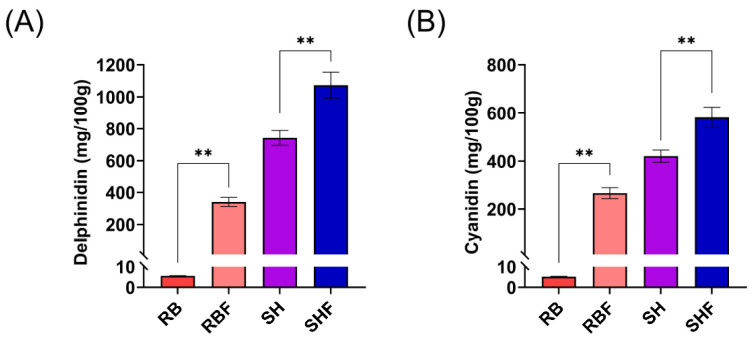
Values of anthocyanins in soluble persimmon fiber-rich fractions. The anthocyanins (**A**) delphinidin and (**B**) cyanidin in mg/100 g persimmon sample (as mean ± SD, n = 3) measured by HPLC, where RB, RBF, are fiber-rich persimmon fractions from Brilliant Red variety, fermented (F) or not; respectively, SH, SHF are fiber-rich persimmon fractions from Sharoni variety, fermented (F) or not; ** *p* < 0.01. RB: Red Brilliant fraction; RBF: Red Brilliant fermented fraction; SH: Sharoni fraction; SHF: Sharoni fermented fraction.

The HPLC analysis demonstrated that the persimmon fiber-rich products contain anthocyanins in abundance, also that the fermentation significantly increased (*p* < 0.01) the amount of extractable delphinidin and cyanidin in both varieties of persimmon (Figure 3), and these anthocyanins are more abundant in the samples from the Sharoni variety. 

### 3.2. Effects of Persimmon Fiber-Rich Fractions on Cell Viability

The effects of the four fractions on cell viability were studied in four cell lines (two of human and two of murine origin) after 24 and 48 h of culture with the fractions. Three concentrations of the fractions were tested (100, 250 and 500 µg/mL). In human cell lines Alamar Blue assay was used. For murine cell lines, the Live/Dead Viability/Cytotoxicity Kit was employed. 

Firstly, none of the different DMSO concentrations equivalent to the amount of vehicle in the corresponding assayed fractions (0.04%, 0.1% and 0.2%) exerted any effect on cell viability.

**Figure 4:**
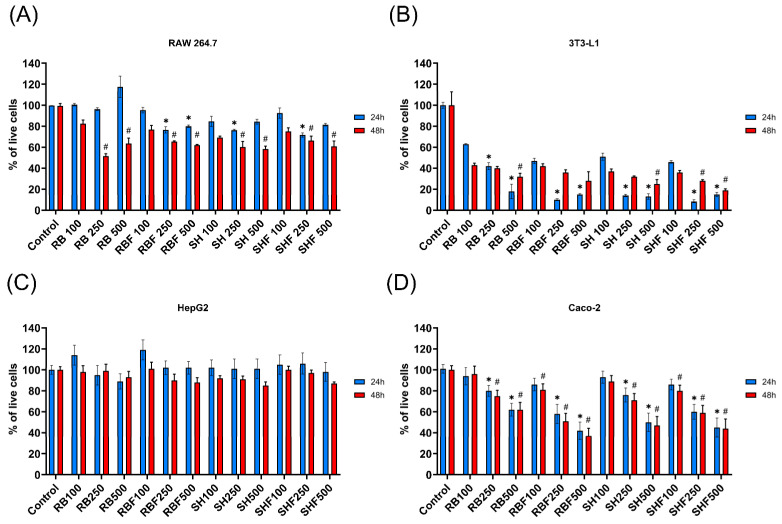
Effect of the different persimmon fiber-rich fractions on the viability on murine and human culture cell lines over 24 and 48 h culture incubations. (**A**) Effect of the fractions on the RAW 264.7 cell line. (**B**) Effect of the fractions on the 3T3-L1 cell line differentiated to adipocytes. (**C**) Effect of the fractions on the HepG2 cell line. (**D**) Effect of the fractions on the Caco-2 cell line. Viability was expressed as a percentage relative to the control (cells cultured without fraction). Blue bars indicate a 24 h culture with the fractions and red bars indicate 48 h culture. The concentrations of the fractions were 100, 250, and 500 µg/mL. Values are expressed as the mean ± SD (n = 4). * *p* < 0.05 fractions versus control at 24 h. # *p* < 0.05 fractions versus control at 48 h. RB: Red Brilliant fraction; RBF: Red Brilliant fermented fraction; SH: Sharoni fraction; SHF: Sharoni fermented fraction.

In all cell lines (Figure 4A–D), the culture with the fractions at 100 µg/mL for 24 h did not affect cell viability. In the 48-hour culture, there was only a significant decrease (*p* < 0.05) in viability with RBF and SHF fractions and only for the Caco-2 cell line (Figure 4D). 

In the RAW 264.7 cell line (Figure 4A), only the RB fraction, at any concentration and during 24 h of culture, did not affect cell viability. In the rest of the culture conditions (type of fraction and duration of culture), there was a significant (*p* < 0.05) decrease in cell viability.

For the HepG2 cell line (Figure 4C), none of the concentrations of the four fractions affected cell viability at 24 h or 48 h of culture with the fractions. At the other extreme was the adipocyte cell line (Figure 4B), where any concentration above 100 µg/mL of all fractions significantly (*p* < 0.05) decreased viability.

In the Caco-2 cell line (Figure 4D), when cultured at concentrations above 100 µg/mL, the four fractions significantly (*p* < 0.05) decreased cell viability at 24 and 48 h of culture.

### 3.3. Anti-Inflammatory Effects of Persimmon Fiber-Rich Fractions

The anti-inflammatory effects were evaluated for the four fractions, but only at concentrations with no cytotoxic effects observed (100 µg/mL). One murine (RAW 264.7) and one human (Caco-2) cell line were studied. Two conditions were tested: (i) previous culture with the fractions for 2 h and then addition of the proinflammatory stimulus for 24 h but without the fractions, and (ii) culture of the fractions and the proinflammatory stimulus together during 26 h. None of the four fractions had proinflammatory effects in the two cell lines, nor did 0.04% DMSO. 

**Figure 5:**
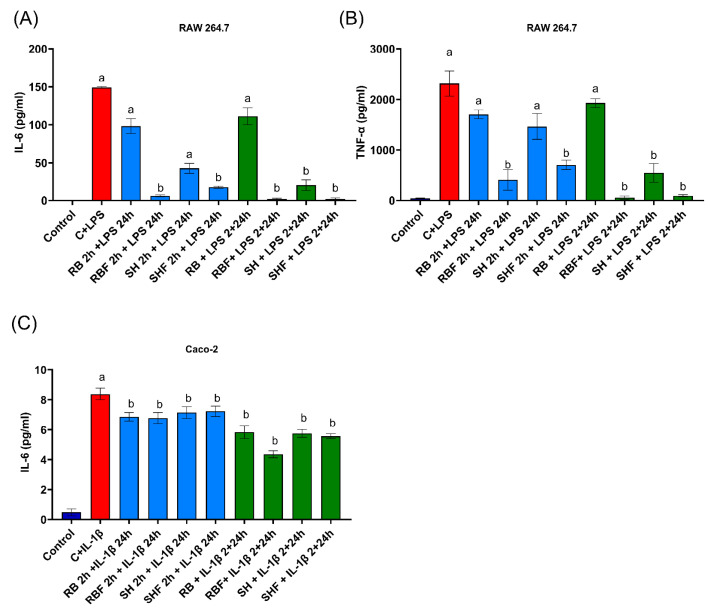
Effects of the different persimmon fiber-rich fractions on inflammation, in murine and human cell lines. (**A**) Effect of the fractions on IL-6 release, in RAW 264.7 cell line. (**B**) Effect of the fractions on TNF-α release, in RAW 264.7 cell line. (**C**) Effect of the fractions on IL-6 release, in Caco-2 cell line. The red bar represents the inflammatory stimulation condition with LPS or IL-1β. The blue bars are the culture conditions with the addition of the fractions before the induction of inflammation and then the addition of LPS (1 µg/mL) or IL-1β (25 ng/mL) with the absence of the fractions. The green bars indicate the simultaneous culture condition of the fractions and LPS (1 µg/mL) or IL-1β (25 ng/mL). The concentration of fraction used was 100 µg/mL. All cultures lasted 24 h. Values are expressed as the mean ± SD (n = 7). Different letters indicate a significant difference (*p* < 0.05) versus induction of inflammation with LPS or IL-1β. RB: Red Brilliant fraction; RBF: Red Brilliant fermented fraction; SH: Sharoni fraction; SHF: Sharoni fermented fraction. LPS: lipopolysaccharide.

In the Caco-2 cell line, all the fractions and in both conditions tested significantly (*p* < 0.05) decreased the inflammatory effect of IL-1β (Figure 5C).

In the RAW 264.7 cell line, RBF and SHF fractions significantly decreased (*p* < 0.05) IL-6 (Figure 5A) and TNF-α (Figure 5B) LPS-induced production in the two conditions tested. The RB fraction had no anti-inflammatory effects. In the case of SH, there was a significant (*p* < 0.05) reduction of IL-6 and TNF-α production in the assay condition of simultaneously culture of fractions and proinflammatory stimulus. 

### 3.4. Effects of Persimmon Fiber-Rich Fractions on the Activity of Antioxidant Enzymes

The antioxidant activity of the four fractions was analyzed in the human line HepG2 at the concentration of the fractions at which viability was not affected (100 µg/mL) and in the same two culture conditions already mentioned in the previous section. 

None of the four fractions had any effect on antioxidant enzyme activity in the HepG2 cell lines, nor did 0.04% DMSO, in the absence of any oxidative stress stimulus.

**Figure 6:**
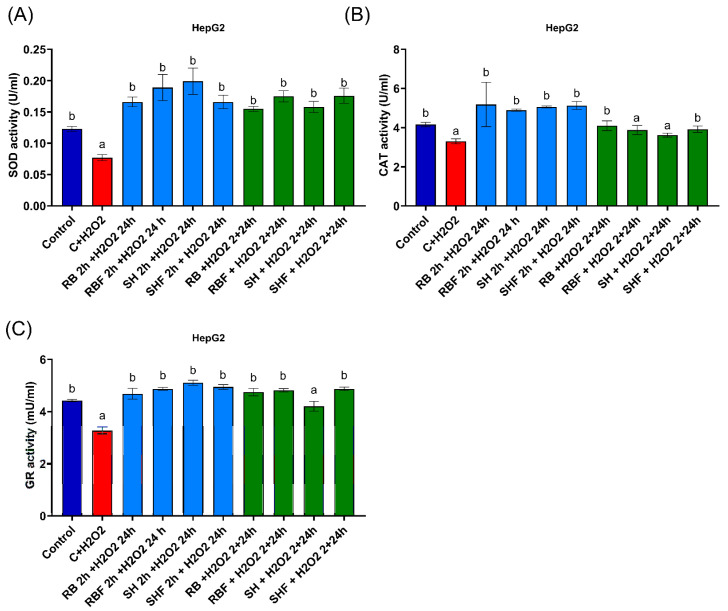
Effects of different persimmon fiber-rich fractions on antioxidant enzyme activity. (**A**) Effect of fractions on SOD activity, in HepG2 line. (**B**) Effect of fractions on CAT activity, in HepG2 line. (**C**) Effect of fractions on GR activity, in HepG2 cell line. The red bar represents the pro-oxidant stimulation condition with H_2_O_2_ (500 μM). The blue bars are the culture conditions with the addition of the fractions before the induction of oxidative stress and then the addition of H_2_O_2_ with the absence of the fractions. The green bars indicate the simultaneous culture condition of the fractions and H_2_O_2_. The concentration of fraction used was 100 µg/mL. All cultures lasted 24 h. Values are expressed as the mean ± SD (n = 7). Different letters indicate a significant difference (*p* < 0.05) versus induction of oxidation with H_2_O_2_. RB: Red Brilliant fraction; RBF: Red Brilliant fermented fraction; SH: Sharoni fraction; SHF: Sharoni fermented fraction. SOD: superoxide dismutase; CAT: catalase; GR: glutathione reductase.

The antioxidant activity of SOD, CAT, and GR enzymes was studied. SOD (Figure 6A) and GR (Figure 6C) enzymes significantly (*p* < 0.05) increased their activity after induction of oxidative damage with H_2_O_2_ when HepG2 cells were cultured with the different fractions and for the two conditions tested. There was only one exception, and that was the cultur.e of SH fractions together with H_2_O_2_ (Figure 6C). In the case of CAT enzyme activity (Figure 6B), there was a significant (*p* < 0.05) increase in its activity for all fractions in the condition of previous culture with the fractions. When RBF and SH fractions and H_2_O_2_ were cultured simultaneously, there was no increase in the activity of the antioxidant enzyme. 

### 3.5. Effects of Persimmon Fiber-Rich Fractions on Bacterial Growth

**Figure 7:**
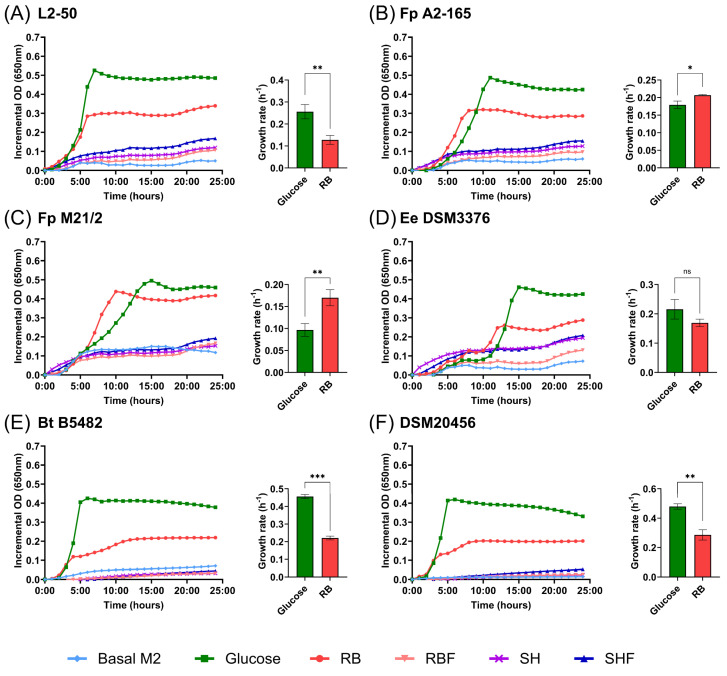
Growth stimulation by RB, RBF, SH, and SHF fiber-rich fractions and comparison of growth rates (h^−1^) between glucose and RB fraction in different bacterial strains. (**A**) *Coprococcus* species L2-50; (**B**) *Faecalibacterium prausnitzii* A2–165; (**C**) *Faecalibacterium prausnitzii* M21/2; (**D**) *Eubacterium eligens* DSM3376; (**E**) *Bacteroides thetaiotaomicron* B5482; (**F**) *Bifidobacterium bifidum* DSM20456. The final fraction concentration was 0.2%. Growth stimulation in microtiter plates values were expressed as mean OD650 values. Values in growth rate were expressed as mean ± SD (n = 3). Basal M2 medium contains no added carbohydrate source. * *p* < 0.05; ** *p* < 0.01; *** *p* < 0.001. ns: not significant. RB: Red Brilliant fraction; RBF: Red Brilliant fermented fraction; SH: Sharoni fraction; SHF: Sharoni fermented fraction.

All strains showed variable, although significant, growth (*p* < 0.01) with the RB fraction (Figure 7), while no significant growth was detected with RBF, SH, and SHF fractions. *Faecalibacterium prausnitzii* strains (A2-165 and M21/2) showed a significantly (*p* < 0.05 and *p* < 0.01, respectively) higher growth rate with RB fraction as a carbohydrate source than with glucose (Figure 7B,C). For the rest of the species, except for *Eubacterium eligens* (Figure 7D), the growth rate with RB fraction as a carbohydrate source was significantly (*p* < 0.01, *p* < 0.001 and *p* < 0.01, respectively) lower than glucose (Figure 7A,E,F). 

**Figure 8:**
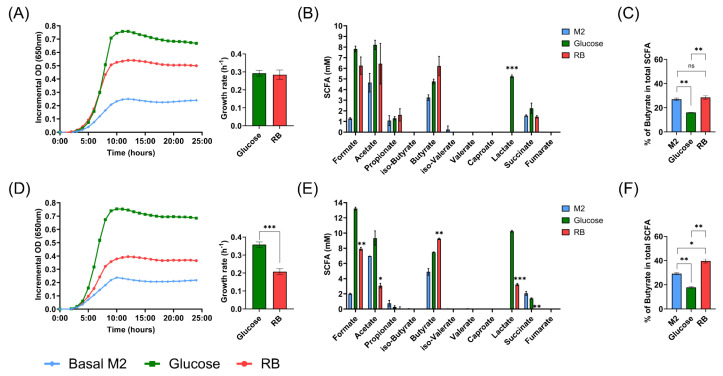
Growth stimulation and short-chain fatty acids production of a mix of three Firmicutes and one Bacteroidetes strain (**A**–**C**) and a mix of two Firmicutes, one Bacteroidetes and one Bifidobacterium strain (**D**–**F**) by glucose and RB fraction. (**A**) Growth rate of three Firmicutes (*Eubacterium eligens* DSM3376; *Faecalibacterium prausnitzii* A2–165; *Faecalibacterium prausnitzii* M21/2) and one Bacteroidetes (*B. thetaiotaomicron* B5482) strains; and (**B**) short-chain fatty acids (SCFA) profile in the medium after a 24 h culture by glucose and RB fraction (n = 2); and (**C**) percentage of butyrate in total SCFA (n = 2). (**D**) Growth rate of two Firmicutes (*Eubacterium eligens* DSM3376, *Faecalibacterium prausnitzii* M21/2), one Bacteroidetes (*B. thetaiotaomicron* B5482) and one Bifidobacteria (*Bifidobacterium bifidum* DSM20456) strains, (**E**) SCFA profile in the medium after 24 h culture by glucose and RB fraction (n = 2) and (**F**) percentage of butyrate in total SCFA (n = 2). Growth stimulation in microtiter plates values were expressed as mean OD650 values. Values in growth rate were expressed as mean ± SD (n = 10). Basal M2 medium contains no added carbohydrate source. mM= millimolar. * *p* < 0.05; ** *p* < 0.01; *** *p* < 0.001. SCFA: short-chain fatty acids; RB: Red Brilliant fraction.

The mix of three Firmicutes strains (*Eubacterium eligens* DSM3376, *Faecalibacterium prausnitzii* A2-165, and *Faecalibacterium prausnitzii* M21/2) and one Bacteroidetes strain (*Bacteroides thetaiotaomicron* B5482) showed equal growth rate with RB fraction and glucose (Figure 8A). In the mix of bacteria, fermentation of the RB fraction resulted in the production of formate, acetate, propionate, butyrate, and succinate at the end of the incubation (Figure 8B), while glucose fermentation resulted in the production of the same SCFA products as the RB fraction plus lactate (Figure 8B). The butyrate level was higher (no significant difference) in RB compared to glucose, while lactate (approx. 5 mM) was only detected when the bacteria were grown on glucose, which is likely due to more rapid growth. The ratio of butyrate as a proportion of the total SCFA was higher (*p* < 0.01) on RB compared to glucose (Figure 8C). 

In a separate experiment with a more complex mix of bacteria, a mix of two Firmicutes strains (*Eubacterium eligens* DSM3376 and *Faecalibacterium prausnitzii* M21/2), one *Bifidobacterium* strain (*Bifidobacterium bifidum* DSM20456), and one Bacteroidetes strain (*Bacteroides thetaiotaomicron* B5482) showed growth with RB fraction, although the growth rate was significantly (*p* < 0.001) lower than glucose (Figure 8D). In the mix of bacteria, fermentation of the RB fraction resulted in the production of formate, acetate, butyrate, and lactate (Figure 8E). Glucose fermentation produced the same SCFA as the RB fraction plus propionate and succinate (Figure 8E). The latter two products are likely to only come from the fermentation activity of the *Bacteroides thetaiotaomicron* strain B5482. In Figure 8E, the butyrate level was significantly higher (*p* < 0.01) in RB compared to glucose. In addition, the percentage of butyrate as a proportion of the total SCFA in RB was twice as much (*p* < 0.01) as in glucose (Figure 8F).

Overall, the RB fermentation results in a more beneficial fermentation profile compared to that of glucose. 

## 4. Discussion

The concentrations of bioactive phytochemicals, such as delphinidin and cyanidin, found in the persimmon fiber-rich fractions, specifically the fermented fractions from the Sharoni variety, are similar to those found in berries [41]. Delphinidin and cyanidin health-promoting benefits such as antioxidant, anti-cancer, anti-inflammatory, cardioprotective and anti-hypertensive are well documented by several reviews [42,43,44]. Berries such as bilberries are particularly rich in anthocyanins and are consumed because of their high content of these bioactives. We also observed that the fermentation used to prepare the persimmon fractions significantly increased the extractability of several bioactive phytochemicals, such as anthocyanins and phenolic acids, suggesting that these molecules could therefore be more bioavailable in vivo and therefore exert health benefits. In fact, the anti-inflammatory effects exerted by the persimmon fiber-rich fractions in murine and human cell lines were higher in both fermented fractions and in the Sharoni variety, which had significantly higher levels of anthocyanins. Regarding the antioxidant activity, we found that all persimmon fiber-rich fractions had antioxidant activity in a human liver cell line. Inflammation and oxidative stress are the most common features of chronic diseases. Functional food ingredients, such as fermented fiber-rich persimmon fractions rich in anthocyanins, could therefore be used in the design of functional foods as part of nutritional therapies to prevent the development of chronic diseases. 

The extracts used in the study were rich in pectin [45]. We found it remarkable that the persimmon pectin fiber-rich fractions in cultured cell lines did not show cytotoxic effects unless they were used at high, non-physiological, and not nutritionally relevant concentrations higher than 100 ug/mL. This agrees with studies showing that pectin can reduce in vitro tumoral cell proliferation and viability in different cell lines from human and murine origins at the same concentrations [46,47]. Several signaling pathways have been proposed for pectin in vitro, with anti-cancer activity being the most relevant to the DNA and mitochondrial damage, the increase in the production of reactive oxygen species, higher levels of apoptosis, cell cycle arrest, and the interference with extracellular matrix proteins [46,47]. Our data show that the most significant effects of the extracts occur in a murine fibroblast cell line and a human colon cancer line. The studies indicate that pectin can reduce in vitro cell viability in some tumor lines more than in others. This occurs regardless of whether the lines are of murine or human origin. No effects of pectin have been described in healthy cells. These results are encouraging as they guarantee their use for the development of food ingredients without having detrimental adverse events in healthy cells and with anti-cancer properties that decrease tumor cell proliferation. Although our results and those of other studies are based on in vitro studies, they open the possibility to further analyze the use of extracts as possible adjuvant therapies against some tumors.

It is, however, important to determine whether industrial by-products from the fruit industry can be used to promote the growth and metabolic output of bacteria in the human colon to promote health as this will help to drive a greener economy and reduce waste by-products from the food industry. The selectivity of pectin-rich food by products, such as those derived from persimmon fruits, for gut bacteria suggest that these fruit by-products could be used as prebiotics. The selectivity of pectin and pectic-oligosaccharides as a growth substrate was demonstrated by Chung et al. [28]. They showed that *Eubacterium eligens* and *Faecalibacterium prausnitzii* utilized pectin and pectic-oligosaccharides as growing substrate. These bacteria showed also to grow very well on model short oligomers (with degree of polymerization of 4 and 5) substrates. Others also showed that a range of whole fruits and vegetables also promoted these important bacterial species [48]. The current study revealed that two *F. prausnitzii* strains (A2-165 and M21/2) grew more rapidly on the none pre-fermented BR substrate than on glucose. One of the other Firmicutes strains of *E. eligens* (DSM3766) showed no significant difference in growth rates between the two substrates, while all the other strains tested achieved lower growth rates on the BR substrate when compared to glucose. When several of the strains belonging to the Bacteroidetes and Firmicutes phyla were mixed together and compared from the growth and fermentation of the none pre-fermented BR compared to glucose, butyrate was one of the major fermentation products formed on the BR substrate, suggesting that this fruit by-product may be a valuable substrate to promote the growth of health-promoting bacteria from the human colon. Moreover, in additional incubations, which included a bacterial strain from a third phylum, *B. bifidum*, it was still evident that butyrate was one of the main fermentation products. These findings are extremely important because of the strains in the mix that only *F. prausnitzii* can form butyrate, and this therefore demonstrates that this bacterium can compete very well for the BR substrate within the strain mixes. *F. prausnitzii* is recognized as one of the most abundant bacterial species in the healthy human colon, in part due to its ability to produce butyrate and also because it has been shown to attenuate the development of colitis in mouse models [49,50]. These bacteria help maintain the integrity of the intestinal mucosa, release molecules with anti-inflammatory properties, and contribute to the maintenance of a healthy microbiota [51]. *F. prausnitzii* also possesses the capacity to induce the anti-inflammatory cytokine interleukin 10 (IL-10) [52], as does the important key pectin degrader *E. eligens* [28]. Butyrate is the major energy source for colonocytes and may modulate carcinogenesis through its effects on proliferation, differentiation, and apoptosis in the gut, as well as stimulation of the immune system [53]. Butyrate also promotes blood flow and gut motility in the colon, which are important for digestion [54]. Furthermore, butyrate supports wound healing processes that are needed to repair epithelial injury [55]. Moreover, butyrate can prevent and treat diet-induced insulin resistance [56]. The crosstalk between the microbiome and host immunity regulates inflammatory status and has an important role in the prevention of chronic non-communicable diseases and health maintenance [57].

The persimmon fruit by-products tested in this study contain a heterogeneous family of phytochemicals with anti-inflammatory, antioxidant, and anti-cancer effects. These effects are most probably due to their richness in anthocyanins. Moreover, the pectin fiber-rich fraction inhibited “in vitro” tumoral cell line proliferation. In addition, the unfermented fractions clearly supported growth of important human colonic bacterial species with respect to promoting the growth of anti-inflammatory and butyrate-producing species that are likely to promote colonic and general health. Thus, fiber-rich fractions, obtained from persimmon fruit by-products can be used to generate foods with healthier properties to be considered as part of new therapeutic resources to promote health. The “in vivo” functionality of new therapeutic foods containing persimmon fiber-rich fractions will be the scope of further investigations.

## Figures and Tables

**Table 1:** Free monosaccharides content in persimmon RB, SH, RBF, and SHF fractions.

Compound (g/100 g)	Fiber-Rich Persimmon Fraction			
	RB	SH	RBF	SHF
Fructose	18.47 ± 0.10 *	8.62 ± 0.92	0 ***	0 ***
Glucose	20.31 ± 1.23 ***	4.83 ± 0.48	0 ***	0 ***

RB: Red Brilliant fraction; RBF: Red Brilliant fermented fraction; SH: Sharoni fraction; SHF: Sharoni fermented fraction. Values are expressed as g/100 g dry weight. Mean ± SD (n = 3). * *p <* 0.05; *** *p* < 0.001.

## Data Availability

The original contributions presented in the study are included in the article, further inquiries can be directed to the corresponding authors.
